# Distribution of candidate genes for experimentally induced arthritis in rats

**DOI:** 10.1186/1471-2164-11-146

**Published:** 2010-03-02

**Authors:** Lars Andersson, Fredrik Ståhl

**Affiliations:** 1 Department of Cell and Molecular Biology-Genetics, Göteborg University, Box 462, SE 40530 Göteborg, Sweden; 2 School of Health Science, University Collage of Borås, SE-501 90 Borås, Sweden

## Abstract

**Background:**

Rat models are frequently used to link genomic regions to experimentally induced arthritis in quantitative trait locus (QTL) analyses. To facilitate the search for candidate genes within such regions, we have previously developed an application (CGC) that uses weighted keywords to rank genes based on their descriptive text. In this study, CGC is used for analyzing the localization of candidate genes from two viewpoints: distribution over the rat genome and functional connections between arthritis QTLs.

**Methods:**

To investigate if candidate genes identified by CGC are more likely to be found inside QTLs, we ranked 2403 genes genome wide in rat. The number of genes within different ranges of CGC scores localized inside and outside QTLs was then calculated. Furthermore, we investigated if candidate genes within certain QTLs share similar functions, and if these functions could be connected to genes within other QTLs. Based on references between genes in OMIM, we created connections between genes in QTLs identified in two distinct rat crosses. In this way, QTL pairs with one QTL from each cross that share an unexpectedly high number of gene connections were identified. The genes that were found to connect a pair of QTLs were then functionally analysed using a publicly available classification tool.

**Results:**

Out of the 2403 genes ranked by the CGC application, 1160 were localized within QTL regions. No difference was observed between highly and lowly rated genes. Hence, highly rated candidate genes for arthritis seem to be distributed randomly inside and outside QTLs. Furthermore, we found five pairs of QTLs that shared a significantly high number of interconnected genes. When functionally analyzed, most genes connecting two QTLs could be included in a single functional cluster. Thus, the functional connections between these genes could very well be involved in the development of an arthritis phenotype.

**Conclusions:**

From the genome wide CGC search, we conclude that candidate genes for arthritis in rat are randomly distributed between QTL and non-QTL regions. We do however find certain pairs of QTLs that share a large number of functionally connected candidate genes, suggesting that these QTLs contain a number of genes involved in similar functions contributing to the arthritis phenotype.

## Background

Rheumatoid arthritis (RA) is a complex inflammatory disease of peripheral joints and is a major cause of disability [1]. Since the disease is dependent on both environmental and genetic factors and since the genetic component is likely to be very complex, the genetics of RA is hard to study [2]. Rat models of RA are therefore frequently used since both the environment and the genetics can be strictly controlled. Several inbred rat strains that are susceptible to experimentally induced arthritis have been established. In these rats a phenotype very much resembling RA in human can be induced by injecting an agent such as collagen, pristane, oil or adjuvant [3]. By conducting genetic linkage analyses of populations derived from arthritis-susceptible and arthritis-resistant rat strains, so called quantitative trait loci (QTLs) can be identified [4]. A QTL can be identified if it harbours a single gene with a strong effect on the quantified phenotype, or several genes with smaller effects on the phenotype. For example, the collagen-induced arthritis QTL Cia5 on rat chromosome 10, has been shown to contain at least three arthritis severity regulating loci [5]. More than 60 QTLs for experimentally induced arthritis in rat have been reported [3,6]. Although these QTLs limit the number of candidate genes, they do in fact cover half of the genome in rat [7]. Taking this rather extensive coverage into account, it seems reasonable to assume that a majority of all genes contributing to the arthritis phenotype should be found within this half of the rat genome. In order to investigate if this assumption is true we explored how candidate genes for experimentally induced arthritis in rat were distributed within and outside arthritis QTLs. This analysis was based on a web tool, Candidate Gene Capture (CGC), that we have previously developed and that ranks candidate genes in QTL regions associated with experimentally induced arthritis in rats [8].

Furthermore, to investigate the hypothesis that certain QTL regions may contain several genes involved in similar functions associated with arthritis, we studied functional connections between genes within collagen-induced arthritis QTL regions from two different rat crosses. Since rats from both these crosses develop similar arthritis phenotypes, they probably have similar biochemical or cellular functions disrupted. The collagen-induced arthritis QTL regions identified in the two rat crosses are very different however. This makes us believe that the unique QTL regions of the two crosses harbour genes involved in similar processes. This hypothesis was investigated by comparing gene functions between different QTLs from the two crosses.

## Methods

To investigate if candidate genes for arthritis are more likely to be found within QTL regions than in non-QTL regions, we conducted a genome wide CGC ranking for 2403 genes with known genomic position in rat and with a CGC score of at least 0.1. Briefly, the CGC application uses an array of 49 preset keywords that have been assigned percentage scores reflecting the connections of the keywords to arthritis. OMIM records for all genes within human regions homologous to an arthritis QTL region in the rat are scanned for the keywords [9]. For each keyword, a so called relevance index was calculated by dividing the number of PubMed abstracts containing both the keyword and the selected reference term with the number of PubMed abstracts containing the keyword alone. The ratio is multiplied by 100 to get the percentage figures [8]. The sum of the keyword scores of all matching keywords for each gene (the CGC score) is used to rank the genes within each QTL. In this study however, the CGC application was modified to rank candidate genes for arthritis genome wide. Gene symbols and gene positions for all of the 2403 genes were collected from RGD and Ensembl [6,10]. The genes were divided into groups based on their individual CGC score. 68 QTL regions were collected from RGD [6], many of which were more or less overlapping (Additional file 1). The number of genes in each CGC score group that were found inside and outside of known arthritis QTLs was then recorded.

To investigate if groups of genes within certain QTLs have functional connections to groups of genes within other QTLs, we analyzed 13 collagen-induced arthritis QTLs in rat. The QTLs were all collected from two arthritis susceptible strains (DA and BB/Dr) crossed with the same arthritis resistant strain (BN) [11,12]. Five QTLs have been identified in the DA × BN cross and nine QTLs have been identified the BB/Dr × BN cross. Only one of these QTLs (Cia13) was found in both crosses. Based on the assumption that the similar phenotype of DA × BN and BB/Dr × BN are caused by gene mutations in their QTL regions, we attempted to find functional connections between genes in QTLs from the two crosses. Using the CGC application we assigned scores to all genes within human regions homologous to the 13 QTLs [8]. To find functional gene pairs between genes in QTLs from the two crosses, the OMIM records for all genes within the 13 QTL regions were scanned for hyperlinks to genes located in a QTL from the opposite cross using the CGC-RefLink application available at the RatMap database [13]. Based on the number of genes within each QTL and the total number of connections between QTLs from the two crosses, Chi-square tests with Yates correction were performed. In this way, QTL pairs with significantly higher numbers of gene connections than what would be expected from the number of genes in each QTL were identified.

To investigate if strong candidate genes within a QTL pair can also be shown to have a strong functional correlation, we selected gene pairs connecting the QTLs where at least one gene had a CGC score of 50 or above. A CGC score of 50 was considered to indicate a gene with a strong functional connection to arthritis. The genes involved in the connections of such a pair of QTLs were considered as a group of functionally related genes. Thereafter, we retrieved common functions for the genes in these groups by using the Gene Functional Classification Tool available at "The Database for Annotation, Visualization and Integrated Discovery " (DAVID) [14,15]. Briefly, the tool generates gene similarity matrices based on shared terms from 14 functional annotation sources (such as Gene Ontology). For each group of genes, functional clusters were created using the medium and highest stringency and genes sharing significant terms were recorded. Higher stringency generates groups with fewer but more tightly associated genes. The geometrical mean of p-scores (Geo) for the terms included in each group was also recorded.

## Results and Discussion

### Genome wide screening for arthritis related candidate genes

To investigate if candidate genes for arthritis are more frequent within experimentally induced arthritis QTLs than in non-QTL regions, we conducted a genome wide CGC ranking for 2403 human genes homologous to rat genes. Out of these 2403 rat genes, we found that 48% were situated within QTL regions and 52% outside QTL regions.

Based on their individual CGC score, the genes were divided into seven groups, where high CGC scores indicate strong connections to arthritis (Table 1). The distribution of genes within and outside QTLs was almost identical in all ranges of CGC scores. Based on these findings, we conclude that candidate genes for arthritis are randomly distributed across the rat genome and evenly distributed between QTL and non-QTL regions. Our conclusion that approximately 50 percent of candidate genes for arthritis genome wide in rat are localized within QTL regions, which do in fact cover half of the rat genome, indicates that there are still many genes with a capacity to contribute to an arthritis phenotype that have not yet been detected through QTL analyses. This is not too surprising however, since a very limited number of rat strains susceptible to arthritis have been used in these studies. Each inbred rat strain has a unique allelic combination, and the experimentally induced arthritis studied using these models are probably just a small subset of the genetic combinations that could lead to an arthritis phenotype. Another explanation for the random distribution between QTLs and non-QTL regions is that the QTL regions are too large, and contain too many genes not involved in the development of the phenotype.

**Table 1:** Candidate genes with different CGC scores located in QTL regions and non-QTL regions in the rat genome.

CGC score	Genes within QTLs	Genes outside of QTLs	P-value from Chi2
200 -	23	22	0.70

100 - 200	23	38	0.10

50 - 100	12	13	0.98

30 - 50	18	16	0.59

20 - 30	59	54	0.40

10 - 20	138	154	0.73

0 - 10	887	946	0.92

Total	1160	1243	

These results are contradictory to the findings recently presented by Xiong et al, who report that 124 out of 185 RA associated genes were located inside QTL regions [7]. The contradicting results of Xiong's group and ours might be explained by the choice of keywords. We both use an automatic selection procedure based on the term "arthritis". Xiong's group use "arthritis" together with a rat gene symbol to select references from PubMed and OMIM, whereas we use the same term to assign values to 49 preselected keywords [8]. After this first selection step, Xiong's group turn to a manual evaluation of the references to select appropriate candidate genes, while our application automatically scans OMIM records for the selected keywords to rank candidate genes. Thus, the principal difference between the method used by Xiong's group and ours is that they use a manual evaluation procedure while ours is automatic. We believe that when conducting an analysis of this kind, the knowledge of established arthritis-associated genes and of the field as a whole, may inadvertently interfere with an unbiased selection of candidate genes. Thus, we believe that an automatic unbiased selection of arthritis-associated genes is to prefer in the type of analysis described above.

### Functional connection between collagen-induced arthritis QTLs in two crosses

In order to find functional connections between different QTLs, we selected data from two different rat strains susceptible to collagen-induced arthritis (DA and BB/Dr) crossed with the same arthritis resistant rat strain (BN). Although animals from both crosses develop a similar phenotype, all QTLs except one (Cia13) are unique to each cross [11,12]. A possible explanation for this difference in QTL distribution may be that genes in QTLs in one strain are involved in the same biochemical pathway or cellular function as genes in QTLs from the other strain. Disruption of reciprocal gene functions associated with arthritis in different QTLs from the two strains could thus result in the same phenotype.

By taking advantage of the hyperlinks between different gene records in OMIM, we were able to connect genes in QTLs from one cross to genes in QTLs from the other cross. We then calculated the expected number of gene pairs shared between every combination of QTLs from the two crosses taking the number of genes in each QTL into consideration. A following Chi-square analysis identified four pairs of QTL regions that all had disproportionally high numbers of gene pairs (P < 0.05): Cia11 - Cia1, Cia11 - Cia2, Cia5 - Cia1 and Cia14 - Cia7 (table 2, Additional file 2). Cia13 - Cia1 with a P value of 0.054 was also included in the further study.

**Table 2:** QTL pairs with a significantly high number of shared gene pairs.

QTL1	Number of genes in QTL1	QTL2	Number of genes in QTL2	Number of gene pairs	P value (Yates)
Cia11	58	Cia1	136	10	0.0017

Cia11	58	Cia2	260	23	7.00E-06

Cia13	135	Cia1	136	18	0.054

Cia5	25	Cia1	136	6	0.018

Cia14	53	Cia7	19	4	0.0024

Out of these five QTL pairs, four shared a number of gene pairs where at least one gene had a CGC score of 50 or above (table 3). The fifth QTL pair (Cia14 - Cia7) shared no gene pairs where at least one gene had a CGC score of 50 or above. The significant connections between gene pairs in QTL regions from the two different crosses may very well be explained by a disruption of reciprocal gene functions associated with arthritis for the four QTL pairs with a number of highly rated candidate genes (CGC score > 50). The same argument also holds for the fifth QTL pair (Cia7 and Cia14), since neither Cia7 nor Cia14 have any genes with a CGC score higher than 50, so the low CGC-score genes are in fact the best candidate genes for these two QTLs.

**Table 3:** QTL pairs with shared gene pairs where at least one has a CGC score of 50 or above.

QTL1	Number of genes in QTL1	QTL2	Number of genes in QTL2	Number of gene pairs >50
Cia11	58	Cia1	136	5

Cia11	58	Cia2	260	10

Cia13	135	Cia1	136	10

Cia5	25	Cia1	136	3

### Functional analysis of the gene pairs connecting QTLs from DA × BN and BB/Dr × BN

Suggesting that the overrepresentation of gene pairs between QTL regions from the two different crosses may be explained by disruption of reciprocal gene functions associated with arthritis, we sought to find such functional similarities by using a web tool (the Gene Functional Classification Tool at DAVID) that groups genes into clusters based on their sharing of sets of functional annotation terms [14,15]. We applied this tool using two levels of stringency, medium and highest, and recorded the level of significance (the Geo-value) and the terms shared. This was done for the gene pairs connecting QTLs where at least one of the genes had a CGC-score of 50 or above. For the QTL pair where no such gene pairs were found (Cia14 - Cia7), all genes connecting the two QTLs where analyzed.

### Cia11 - Cia1

With medium stringency we capture all gene pairs that were available for analysis in DAVID into one single cluster. The Geo-value was significantly low for the cluster as whole. There were six terms included in the cluster of which four were related to protein binding complex, such as "protein complex" and "protein binding".

The same genes were also included in a second cluster but with a non-significant Geo-value (0.11). The cluster was composed of 14 terms, most of which related to plasma membrane structures and functions. Three of the terms referred to immune system processes. All these terms had a significant P-value and altogether involved four of the genes.

Examples of gene pairs between Cia11 and Cia1 where both could give rise to similar phenotypic effects associated with arthritis are B2M - TAP1 and CD44 - TNF. Both TAP1 (Cia1) and B2M (Cia11) are essential for proper antigen presentation on MHC class 1, TAP1 by translocating peptides from the cytosol to the awaiting MHC and B2M by being required for the stable cell-surface expression of MHC class 1 [16,17]. CD44 (Cia11) acts as a negative modulator of TNF-induced joint destruction and inflammatory bone loss, phenotypes that are typical for both RA and collagen-induced arthritis [18].

### Cia11 - Cia2

With medium stringency, all 15 genes tested were present in one or more of six significant clusters. For each single cluster, the genes were more or less evenly distributed from the two QTL-regions. The six clusters were built up from a total of 46 terms, some of which have a clear connection to arthritis, such as "immunoglobulin domain" and "inflammatory response".

Even with the highest stringency for clustering (meaning that all genes in a cluster share all terms included) we found two significant clusters that contain nine and eight genes respectively, rather evenly distributed between the two QTL-regions.

Examples of gene pairs between Cia11 and Cia2 where both could give rise to similar phenotypic effects associated with arthritis are CD44 - TGFB1 and B2M - FCGRT. CD44 (Cia11) is an activator of TGFB1 (Cia2), which in turn downregulates inflammation [19]. B2M (Cia11) together with FCGRT (Cia2), a nonclassical MHC class 1 alpha chain, makes up the neonatal Fc receptor FcRn, which transports IgG across polarized epithelial cells and protects IgG from degradation in the phagocytotic vacuole [20]. FcRn is expressed in monocytes, macrophages and dendritic cells [21].

### Cia13 - Cia1

With medium stringency all genes tested except one on Cia13 (TNFRSF7) built up a very large cluster (Geo-value 0.0094). The cluster contained 102 terms of which many indicated an immunological response ("TGFBr - signalling", "response to stress", "defence response", "response to wounding", response to external stimulus", "inflammatory response").

When using the highest clustering stringency we found eight significant clusters. Many of these clusters shared terms with a potential connection to arthritis. One of the clusters contained all of the genes from the medium stringency cluster.

An example of gene pairs between Cia13 and Cia1 where both could give rise to similar phenotypic effects associated with arthritis is TNF - TNFRSF1A. When TNF (Cia1) is produced by macrophages and T-cells in response to infection, it can bind to and function through one of its receptors, TNFRSF1A (Cia13). Activation of TNFRSF1A can lead to activation of NF-kB, a transcription factor that can induce expression of a large array of genes regulating the immune system. On the other hand, binding of TNF to TNFRSF1A can also lead to apoptosis [22].

### Cia5 - Cia1

With medium stringency, no significant cluster was presented. However, the three included genes ACE, TNF and ICAM2 (one gene was not included) all shared the term "plasma membrane" with significant P-value (P = 0.022).

An example of gene pairs between Cia5 and Cia1 where both could give rise to similar phenotypic effects associated with arthritis is TNF - ICAM2. ICAM2 (Cia5) can protect B-cells from TNF-mediated apoptosis [23]. TNF (Cia1) induces activation of several proinflammatory mediators and ICAM2 has been identified as a potential therapeutic target to inhibit fibroblast-like synoviocyte-activation in RA [24].

### Cia14 - Cia7

With medium stringency, two clusters were found with significant Geo-scores. All four genes included in the search were present in both groups. The first group contained terms like "receptor binding" and several terms referring to positioning of proteins in the cell, like "establishment of protein localization", "macromolecule localization" and "cellular localization". The second group also contained "receptor binding". In addition, the second group contained terms like "cytokine activity", "positive regulation of cell proliferation" and "extracellular space".

Using the highest stringency, three groups with significant Geo-values were found. The first group, containing three genes, included terms referring to protein localization. The second group, containing all four genes, included terms describing protein localization. The third group, containing three genes, included 23 terms like "cytokine activity", "positive regulation of cell proliferation" and "extracellular space".

In summary, when investigating all gene pairs from the significantly connected QTL regions of the two crosses (DA × BN and BB/r × BN) for functional similarities, we do find significant correlations. Thus, using the DAVID application, for four of the five pairs of significantly connected QTLs we found clusters of genes that share different terms more or less associated with arthritis. For the fifth pair of connected QTLs (Cia5 - Cia1) we did not find any cluster but all three genes shared one term of significant value.

Our findings of significantly connected QTLs that also share gene clusters with terms associated with arthritis, support the idea that genes in QTLs from one rat cross are involved in the same biochemical pathway or cellular functions as genes in QTLs from the other cross. The results also suggest that the QTLs in the functionally connected QTL pairs contain several genes contributing to the arthritis phenotype rather than one gene with a strong effect.

## Conclusions

Based on the result from the genome wide CGC search, we conclude that candidate genes for arthritis in rat are randomly distributed between QTL and non-QTL regions. This is in contrast to results presented by others, but we believe that the automatic ranking procedure of the CGC application provides a more unbiased selection of candidate genes, which in turn render a more reliable estimation of gene distribution.

The study of functional connections resulted in five cases where significantly high numbers of gene pairs were found between specific QTLs from the two crosses. Furthermore, the functional studies of the genes connecting two such QTLs show that it is possible to find functions in common for a majority of those genes. These findings suggest that the functions shared by genes in two connected QTLs might be disrupted in animals from both rat crosses and in this way contribute to the arthritis phenotype.

Finally, the findings that several genes involved in similar functions are present within a significantly connected pair of QTLs, and that these functions can be associated with arthritis, indicate that these QTLs harbour more than one gene with effects on the arthritis phenotype.

## Competing interests

The authors declare that they have no competing interests.

## Authors' contributions

LA carried out the analysis, contributed with original ideas and drafted the manuscript. FS supervised the project, contributed with original ideas and took part in the preparation of the manuscript. Both authors read and approved the final manuscript.

## Supplementary Material

Additional file 1 **QTLs included in the study of distribution of candidate genes**. Contains a list of all QTL regions included in the study, including QTL symbol, LOD score, P value, chromosome number, start position and stop position. Click here for file

Additional file 2 **Genes interconnecting the five investigated QTL pairs**. Displays the genes that connect the QTLs in table 2 and the individual CGC score of each gene. Click here for file
