# Supplementary material for: Distribution of candidate genes for experimentally induced arthritis in rats
Source: BMC Genomics. 2010 Mar 2;11:146. doi: 10.1186/1471-2164-11-146 (PMC2838850; doi:10.1186/1471-2164-11-146)
Supplement: Additional file 1 — QTLs included in the study of distribution of candidate genes. Contains a list of all QTL regions included in the study, including QTL symbol, LOD score, P value, chromosome number, start position and stop position. [file 1471-2164-11-146-S1.DOC]

**Additional file 1.** A list of the QTLs included in the study of distribution of candidate genes, including QTL symbol, LOD score, P value, chromosome number, start position and stop position.

| QTL symbol | **LOD score** | **P value** | **Chromosome** | **Start (bp)** | **Stop (bp)** |
| --- | --- | --- | --- | --- | --- |
| Pia8 | 4.7 |  | 1 | 0 | 64145956 |
| Pia11 | 5.3 |  | 1 | 112480604 | 157480604 |
| Cia2 | 5.0 |  | 1 | 33231434 | 78231434 |
| Cia7 | 4.6 | .0001 | 2 | 59700886 | 146659672 |
| Cia10 | 3.4 |  | 2 | 188136088 | 233136088 |
| Cia11 | 5.6 |  | 3 | 26674018 | 97524000 |
| Pia2 | 3.9 |  | 4 | 17617956 | 60974511 |
| Aia2 | 5.8 |  | 4 | 36592628 | 73972591 |
| Pia5 | 4.5 |  | 4 | 60974346 | 130228614 |
| Aia3 | 3.9 |  | 4 | 73972453 | 133939659 |
| Pia9 |  | .048 | 4 | 81916057 | 126916057 |
| Cia3 | 4.8 |  | 4 | 104415843 | 149415843 |
| Ciaa4 | 3.1 |  | 4 | 128179957 | 171204531 |
| Pia23 | 5.3 |  | 4 | 133939407 | 171204531 |
| Pia7 | 4.3 |  | 4 | 133939407 | 171204531 |
| Cia13 | 4.5 |  | 4 | 134858561 | 171204531 |
| Oia2 |  | .001 | 4 | 136668162 | 181668162 |
| Oia8 |  |  | 4 | 136668162 | 181668162 |
| Cia24 | 4.5 |  | 4 | 149304432 | 179854843 |
| Oia7 |  | .0001 | 4 | 159298584 | 160528447 |
| Ciaa5 | 3.5 |  | 5 | 99080786 | 149757440 |
| Pia16 | 3.9 |  | 6 | 47039212 | 108621490 |
| Pia3 | 4.5 |  | 6 | 98840516 | 144200901 |
| Pia24 |  |  | 6 | 117361909 | 147636619 |
| Cia4 | 5.3 |  | 7 | 21202126 | 66202126 |
| Pia17 | 4.7 |  | 7 | 78497123 | 137838800 |
| Pia18 | 4.0 |  | 8 | 79347688 | 87122814 |
| Cia6 |  |  | 8 | 86627869 | 127907359 |
| Cia15 | 4.6 |  | 9 | 2948882 | 39821284 |
| Ciaa3 | 6.5 |  | 9 | 18347567 | 63347567 |
| Cia16 | 3.2 |  | 10 | 6380753 | 100633982 |
| Cia21 | 3.1 |  | 10 | 14721135 | 110718848 |
| Aia5 |  | .01 | 10 | 24006429 | 110718848 |
| Cia20 | 3.2 |  | 10 | 24006429 | 110718848 |
| Pia10 |  | .01 | 10 | 24006429 | 110718848 |
| Pia15 | 3.1 |  | 10 | 24731630 | 47777990 |
| Pia26 |  |  | 10 | 35131252 | 76591208 |
| Cia29 |  |  | 10 | 35131252 | 95695218 |
| Cia22 | 8.9 |  | 10 | 41339689 | 110718848 |
| Cia23 | 3.9 |  | 10 | 41339689 | 110718848 |
| Ciaa2 | 7.1 |  | 10 | 64308668 | 95695218 |
| Oia4 |  |  | 10 | 79229067 | 91168491 |
| Oia5 |  |  | 10 | 91168332 | 110718848 |
| Oia3 |  | .001 | 10 | 91455572 | 110718848 |
| Cia5 | 4.9 |  | 10 | 95692240 | 110718848 |
| Cia27 |  |  | 10 | 95695048 | 110718848 |
| Cia28 |  |  | 10 | 100633512 | 110718848 |
| Pia25 |  |  | 10 | 100633512 | 110718848 |
| Oia6 |  |  | 10 | 101532360 | 108776963 |
| Cia25 | 4.7 |  | 12 | 0 | 43432656 |
| Cia12 | 4.6 |  | 12 | 0 | 44521457 |
| Pia22 | 4.1 |  | 12 | 530865 | 45530865 |
| Pia12 | 8.9 |  | 12 | 555108 | 24748499 |
| Pia20 | 3.6 |  | 14 | 10340377 | 41608939 |
| Pia6 | 4.9 |  | 14 | 20165393 | 45020354 |
| Pia13 | 3.3 |  | 14 | 43399833 | 105009429 |
| Aia4 | 3.0 |  | 15 | 61458810 | 99039586 |
| Pia14 | 4.4 |  | 16 | 59398206 | 88891349 |
| Cia17 | 4.6 |  | 18 | 51166414 | 87265094 |
| Cia26 | 3.6 |  | 18 | 57870442 | 87265094 |
| Pia19 | 3.4 |  | 18 | 77452390 | 82920522 |
| Cia14 | 3.0 |  | 19 | 24304442 | 45692656 |
| Pia21 | 3.3 |  | 20 | 0 | 4740814 |
| Cia1 |  |  | 20 | 0 | 14605852 |
| Aia1 | 18.0 | .001 | 20 | 2790738 | 4531120 |
| Ciaa1 | 30.0 | .001 | 20 | 2790738 | 4531120 |
| Cia19 | 4.4 |  | X | 0 | 88514313 |
| Cia18 | 3.1 |  | X | 4384157 | 49384157 |
