# Supplementary material for: Distribution of candidate genes for experimentally induced arthritis in rats
Source: BMC Genomics. 2010 Mar 2;11:146. doi: 10.1186/1471-2164-11-146 (PMC2838850; doi:10.1186/1471-2164-11-146)
Supplement: Additional file 2 — Genes interconnecting the five investigated QTL pairs. Displays the genes that connect the QTLs in table 2 and the individual CGC score of each gene. [file 1471-2164-11-146-S2.DOC]

**Additional file 2.** A list of genes interconnecting the five investigated QTL pairs and the individual CGC score of each gene. Gene pairs where at least one of the genes received a CGC score of 50 or above are shaded in the table.

| **Cia11** | |  | **Cia1** | |
| --- | --- | --- | --- | --- |
| CD44 | 233.7 | - | TNF | 383.4 |
| PCNA | 2.8 | - | CDKN1A | 242.4 |
| TP53BP1 | 5.3 | - | CDKN1A | 242.4 |
| PLA2R1 | 8.8 | - | TNF | 383.4 |
| B2M | 23.5 | - | TAP1 | 117.7 |
| B2M | 23.5 | - | TAPBP | 30.4 |
| CDC25B | 0 | - | MAPK14 | 24.6 |
| WT1 | 7.7 | - | BCR | 2.8 |
| B2M | 23.5 | - | CSTB | 0 |
| GCG | 12.5 | - | GLP1R | 0 |
|  |  |  |  |  |
| **Cia11** | |  | **Cia2** | |
| TYRO3 | 244.9 | - | AXL | 244.9 |
| AVP | 15.3 | - | ESR1 | 121.3 |
| OXT | 0 | - | ESR1 | 121.3 |
| CD44 | 233.7 | - | TGFB1 | 128.2 |
| ITGB6 | 6.8 | - | TGFB1 | 128.2 |
| ACVR1 | 0 | - | TGFB1 | 128.2 |
| GCG | 12.5 | - | VIP | 233.6 |
| B2M | 23.5 | - | FCGRT | 131.8 |
| CD44 | 233.7 | - | HAS1 | 24.2 |
| CD44 | 233.7 | - | VIL2 | 9.7 |
| PRNP | 25 | - | SOD2 | 10.6 |
| PRNP | 25 | - | PLG | 22.3 |
| BDNF | 9.6 | - | PLG | 22.3 |
| SN | 15.8 | - | CD22 | 17.5 |
| PRNP | 25 | - | BAX | 21.5 |
| PRNP | 25 | - | FPRL1 | 11.6 |
| CD59 | 12.5 | - | PLAUR | 9.2 |
| CKMT1 | 0 | - | CKM | 6.2 |
| SN | 15.8 | - | SIGLEC8 | 5.8 |
| TBR1 | 0 | - | T | 5.5 |
| SN | 15.8 | - | CD33 | 2.4 |
| PCNA | 2.8 | - | POLD1 | 2.4 |
| BDNF | 9.6 | - | GRIN2D | 0 |

|  |  |  |  |  |
| --- | --- | --- | --- | --- |
| **Cia13** | |  | **Cia1** | |
| CDKN1B | 10.6 | - | CDKN1A | 242.4 |
| TNFRSF1A | 55.9 | - | TNF | 383.4 |
| LAG3 | 13.8 | - | TNF | 383.4 |
| TNFRSF7 | 12.7 | - | TNF | 383.4 |
| SIAT8A | 2.8 | - | TNF | 383.4 |
| OLR1 | 5.8 | - | TNF | 383.4 |
| DDX11 | 2 | - | TNF | 383.4 |
| VWF | 34.8 | - | CYP21A2 | 50 |
| LTBR | 107.5 | - | LTB | 2.5 |
| TNFRSF1A | 55.9 | - | LTB | 2.5 |
| A2M | 5.8 | - | C4B | 43.3 |
| AICDA | 9.5 | - | PIM1 | 18.4 |
| PPARG | 21.2 | - | RXRB | 15.2 |
| PPARG | 21.2 | - | PPARD | 12.7 |
| GPR19 | 0 | - | DRD1 | 2.8 |
| VWF | 34.8 | - | BCR | 2.8 |
| PTPN6 | 14 | - | G6B | 0 |
| ETV6 | 4.6 | - | TEL2 | 0 |
|  |  |  |  |  |
| **Cia5ab** | |  | **Cia1** | |
| ACE | 3.6 | - | TNF | 383.4 |
| ICAM2 | 10.2 | - | TNF | 383.4 |
| GH1 | 4.8 | - | TNF | 383.4 |
| CD79B | 8.3 | - | PIM1 | 18.4 |
| MAP2K6 | 0 | - | MAPK14 | 24.6 |
| FALZ | 0 | - | HMGIY | 6.2 |
|  |  |  |  |  |
| **Cia14** | |  | **Cia7** | |
| IL15 | 27.3 | - | IL2 | 21.4 |
| NFATC3 | 11.3 | - | IL2 | 21.4 |
| IL15 | 27.3 | - | IL21 | 25.7 |
| GLG1 | 2.8 | - | FGF2 | 3.4 |
